# Shifts in the Distribution of Mass Densities Is a Signature of Caloric Restriction in *Caenorhabditis elegans*

**DOI:** 10.1371/journal.pone.0069651

**Published:** 2013-07-29

**Authors:** Alfonso Reina, Anand Bala Subramaniam, Anna Laromaine, Aravinthan D. T. Samuel, George M. Whitesides

**Affiliations:** 1 Department of Chemistry and Chemical Biology, Wyss Institute for Biologically Inspired Engineering, Harvard University, Cambridge, Massachusetts, United States of America; 2 Department of Physics and Center for Brain Science, Harvard University, Cambridge, Massachusetts, United States of America; Harvard University, United States of America

## Abstract

Although the starvation response of the model multicellular organism *Caenorhabditis elegans* is a subject of much research, there is no convenient phenotypic readout of caloric restriction that can be applicable to large numbers of worms. This paper describes the distribution of mass densities of populations of *C. elegans*, from larval stages up to day one of adulthood, using isopycnic centrifugation, and finds that density is a convenient, if complex, phenotypic readout in *C. elegans.* The density of worms in synchronized populations of wildtype N2 *C. elegans* grown under standard solid-phase culture conditions was normally distributed, with distributions peaked sharply at a mean of 1.091 g/cm^3^ for L1, L2 and L3 larvae, 1.087 g/cm^3^ for L4 larvae, 1.081 g/cm^3^ for newly molted adults, and 1.074 g/cm^3^ at 24 hours of adulthood. The density of adult worms under starvation stress fell well outside this range, falling to a mean value of 1.054 g/cm^3^ after eight hours of starvation. This decrease in density correlated with the consumption of stored glycogen in the food-deprived worms. The density of the worms increased when deprived of food for longer durations, corresponding to a shift in the response of the worms: worms sacrifice their bodies by retaining larvae, which consume the adults from within. Density-based screens with the drug Ivermectin on worms cultured on single plates resulted in a clear bimodal (double-peaked) distribution of densities corresponding to drug exposed and non-exposed worms. Thus, measurements of changes in density could be used to conduct screens on the effects of drugs on several populations of worms cultured on single plates.

## Introduction

The nematode *Caenorhabditis elegans* is well-established as a model multicellular organism for studying eukaryotic metabolism due, in part, to its short generation time, ease of culture, and amenity to genetic manipulation [1], [2], [3]. Landmark studies conducted on *C. elegans* have revealed the intimate connection between lifespan and metabolism [4], [5], [6], [7], [8], [9], the importance of insulin-like signaling for regulation of fat levels and obesity [6], [10], [11], and the effects of neurotransmitters, such as serotonin [12], and drugs, such as the antidepressants mianserin and methiothepin, on the accumulation and breakdown of fat [13], [14]. Experiments that subjected worms to medium term (on the order of days) food deprivation, before assaying for effects on signaling cascades [5], [15], [16], [17], osmoregulation [18], [19], macronutrient consumption [10], [20], metabolic rate [10], [16], [21], [22], diapause/hibernation [11], [18], [23], lifespan [4], [5], [6], [7], [8], [24], and reproductive capacity [25], [26], [27] have been particularly illuminating.

Current metabolic assays in C. *elegans* typically employ techniques such as optical observation of the spatio-temporal partitioning of fluorescent molecules [10], [21], [22], [28], [29], [30], label-free observation of lipids using spectroscopic techniques [31], observation of changes in the behavior of the worm, such as pumping rate [32] or movement [33], observation of changes in the lifespan [4], [5], [6], [7], [8], [24], and observation of changes in the biochemical composition using techniques such as gas chromatography/mass spectrometry (GCMS) [28], [34], or enzyme-based colorimetric assays [13], [35]. In principle, measuring changes in a physical parameter (i.e. mass, length, or volume) [35], [36] has the potential to be a non-invasive probe of the properties of the worms; in practice, it is difficult to measure these values statistically in a population of worms.

Density– the mass per unit volume– has the potential to be an accessible, quantitative parameter, since the density of large numbers of small objects can be measured easily by isopycnic centrifugation in density gradients. Indeed, the density of the organism should reflect composition: lipid/fat, protein, sugars, salts, and water, have specific densities. Normal development and aging, and adaptation to external stresses such as starvation, or exposure to chemicals, should manifest as changes in net density due to changes in the ratio of these substances in the worms. Thus, assays that measure changes in the mass density of the worms could be a useful addition to the tools used to study this animal. Measurements of density in single-component liquids (i.e. not in density gradients) have been used previously to determine the adiposity (the relative fat content) of large mammals such as humans [37], [38], and also recently of small model organisms, such as Dropsophila larvae [39].

In this paper, we use isopycnic density centrifugation in Percoll™ to measure quantitatively the distribution of mass densities in populations of *C. elegans*. We found that the densities of worms in synchronized populations were normally distributed. We established baseline values of the density of wild-type *C. elegans* as it progressed through the four larval stages and for the first 24 hours of adulthood under standard laboratory solid-phase culture conditions. The distribution of densities in a population of *C. elegans* was sharp for each life-stage (the standard deviation of the normal distribution ranged from 0.002–0.004 g/cm^3^). This distribution suggests that the composition of the worms changes relatively uniformly when grown under ideal conditions.

We measured the distribution of densities of day 1 adult hermaphrodite worms (within the first 24 hours after the L4 molt) subjected to short-term (less than 24 hours) deprivation of food. These experiments are complementary to reports of the effects of starvation on worms in the literature [10], [16], [22], [24]. Our experiments showed that the average density of the worms decreased significantly during the first eight hours of starvation. This decrease was contrary to our naïve expectation that the net density of the worms would increase due to the consumption of lipid stocks (the density of lipids are lower than that of water and most other molecules in an organism [37], [38]). Worms grown on plates with ample food did not undergo a similar decrease in density. Colorimetric assays of the worms revealed that the worms consume glycogen during the initial stages of starvation; the decrease in glycogen correlated with the measured decrease in density. After eight hours, the density of the worms increased, primarily due to the development of larval stages, which have higher densities, in the starving adults (facultative matricide or bag-of-worms phenotype [25], [40]). The width of the distribution increased with the duration of starvation, which might reflect individual differences in adapting to starvation stress. Reintroduction of food after short bouts of starvation resulted in a shift back to pre-starvation densities, and a return to pre-starvation levels of glycogen.

Since changes in density proved a reliable indicator of early stage starvation in adult wild type worms, as proof of principle, we conducted screens with the drug Ivermectin, which prevents normal feeding by paralyzing the pharyngeal muscles in the worm [32], [33]. We find a clear bimodal (double-peaked) distribution of densities in our screens corresponding to drug exposed and non-exposed worms.

## Results and Discussion

### Cold immobilized *C. elegans* form distinct bands when centrifuged in Percoll™ centrifugation media

**Figure 1:**
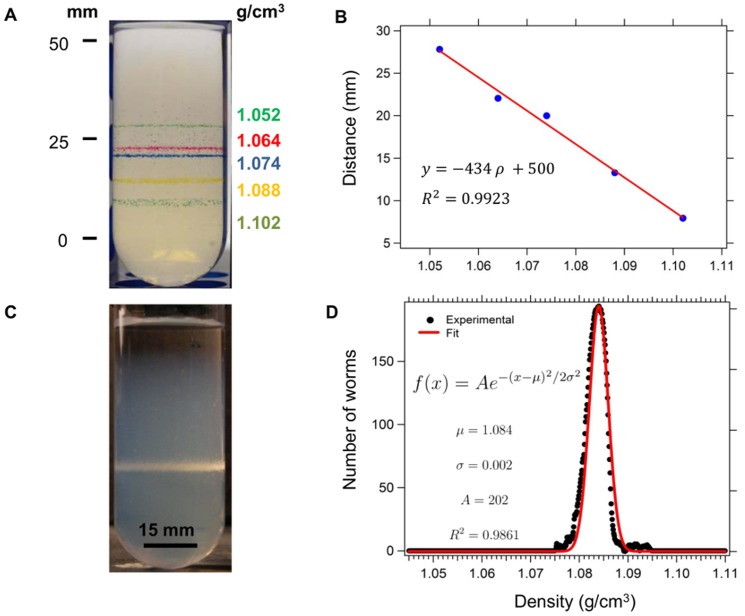
The distribution of mass densities of populations of *C.elegans* can be measured through isopycnic centrifugation in linear Percoll™ gradients. (A) Image of a typical Percoll™ centrifugation medium, with density-standard beads. (B) Plot of the height of the beads versus their intrinsic densities. Each point in the plot is an average of seven measurements, error bars are within the size of the points on the graph. (C) Typical image of cold-immobilized worms, (here, immediately after the L4/adult molt), after centrifugation in the gradient. (D) Distribution of densities of the worms in (C). The red line shows a Gaussian fit to the data.

We prepared centrifugation media from phosphate-buffered saline (PBS) and the commercial density gradient medium Percoll™ in 50-mL polycarbonate centrifuge tubes, as described in the Materials and Methods section. We centrifuged standard beads, of known densities (1.102 g/cm^3^, 1.088 g/cm^3^, 1.074 g/cm^3^, 1.064 g/cm^3^, 1.052 g/cm^3^), in the centrifugation media to characterize the distribution of densities as a function of height (defined as linear distance from the bottom of the tube). **Figure 1** **A** shows a typical image of the equilibrium height of the beads in the media. **Figure 1** **B** shows a plot of the height (averaged over seven replicates) of the beads versus their intrinsic densities in the centrifugation media. The positions of the beads, and thus the gradient of densities produced in the centrifugation media were very reproducible with a standard deviation in height of ∼±0.3 mm (±0.0007 g/cm^3^). It is apparent that the height of the beads in the media varied linearly with their density. By fitting a straight line to our data, we obtained a calibration curve of density versus height in the centrifugation media (**Figure 1** **B**).

**Figure 1** **C** shows a typical image after isopycnic centrifugation in the centrifugation media of live cold-immobilized N2 worms harvested and measured immediately after the L4/adult molt. The worms settled at an equilibrium height that corresponded to their density. **Figure 1** **D** shows the distribution of densities of this population, obtained after analysis from line intensity profiles of the image (See Materials and Methods for details). The densities were normally distributed and peaked sharply at 1.084 g/cm^3^ at this stage of the lifecycle (**Figure 1** **D**). The centrifugation process did not appear to harm the worms as all the worms isolated from the media after centrifugation resume regular movement and feeding activity.

### The mass densities of well-fed *C. elegans* varied significantly between larval and adult stages

**Figure 2:**
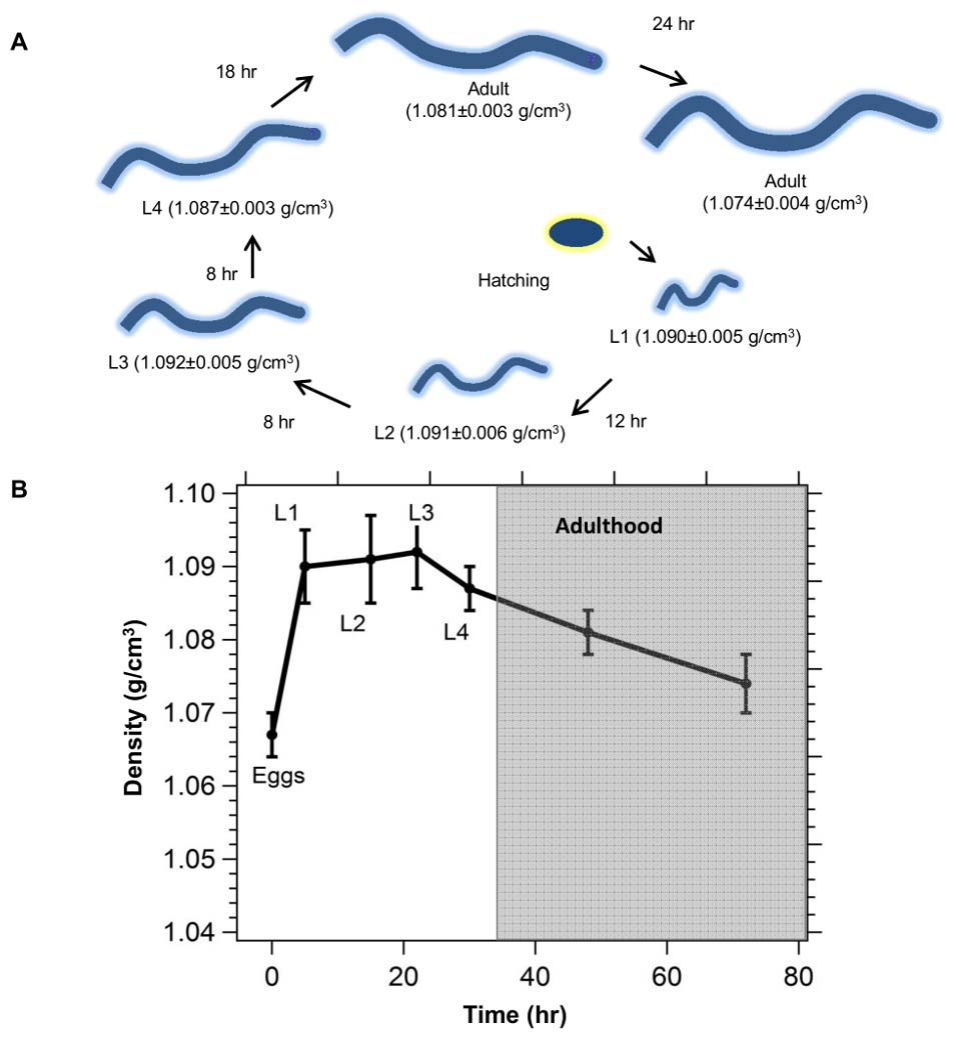
Density is a function of the developmental stage of *C. elegans*. (A) Schematic of the change in density as a function of life cycle. (B) Plot of the mean density of the worms versus time with the bars in the plot indicating one standard deviation of the normal distribution that was fitted to the density distribution curves of a representative population worms at each life stage.

We measured the distribution of densities of populations of worms at each stage of lifecycle, from hatching to the end of the first day of adulthood, to determine if there was any relation between mass density and life stage in *C. elegans*. Distributions of densities were measured on several hundreds of worms from at least three different agar plates to obtain sample sizes that were representative of a population at each stage of the lifecycle. The mean density of L1, L2, and L3 worms were relatively high, and did not vary significantly with each molt. The mean densities were peaked sharply at 1.091 g/cm^3^ with standard deviations of ∼0.004 g/cm^3^ for these three larval stages, i.e. ∼68.3 % of the worms had densities between 1.087 and 1.095 g/cm^3^, and 99.7 % of the population lie between 1.079 and 1.103 g/cm^3^. At the L3/L4 molt, the mean density of the worms decreased slightly to 1.087 g/cm^3^. After the final larval molt, the mean density of the worms fell to 1.081 g/cm^3^ for the newly molted adults. Over the course of the first 24 hours of adulthood, the mean density of the worms decreased significantly to 1.074 g/cm^3^ (**Figure 2**). Thus, the density of the worms decreased with age, which can be seen in the plot of density versus time shown in **Figure 2** **B**, with the bars in the plot indicating one standard deviation of the normal distribution.

The apparent decrease in density as the worms exited the larval stages and entered adulthood could possibly be due to the decrease in the ratio of the protein-rich cuticle to cellular mass and volume as the worm matured [41]. Another potential explanation is the accumulation of fertilized lipid-rich eggs during the first day of adulthood [42]. If the mass density of the eggs were low, accumulation of the eggs in the body of the worms, assuming everything else equal, should lead to a decrease in the net density of the worms. We measured the density of *C. elegans* eggs and found that the density of the eggs was peaked at 1.067 g/cm^3^ with a standard deviation of 0.003 g/cm^3^ (**Figure 2** **B** **, S1**). This result is consistent with the observation that the mean density of egg-containing adults was lower than those of immature larvae. A further potential test of this hypothesis is to use germ-free lines or adult males [10]. Although not attempted here, the large difference in the mean densities of adult and larval worms (∼0.01–0.02 g/cm^3^) could be exploited to separate these two stages in mixed populations by centrifugation. Clear separation by centrifugation of the four larval stages from each other, or adult stages from each other, is likely not possible.

### Adult worms deprived of food decreased in mass density

**Figure 3:**
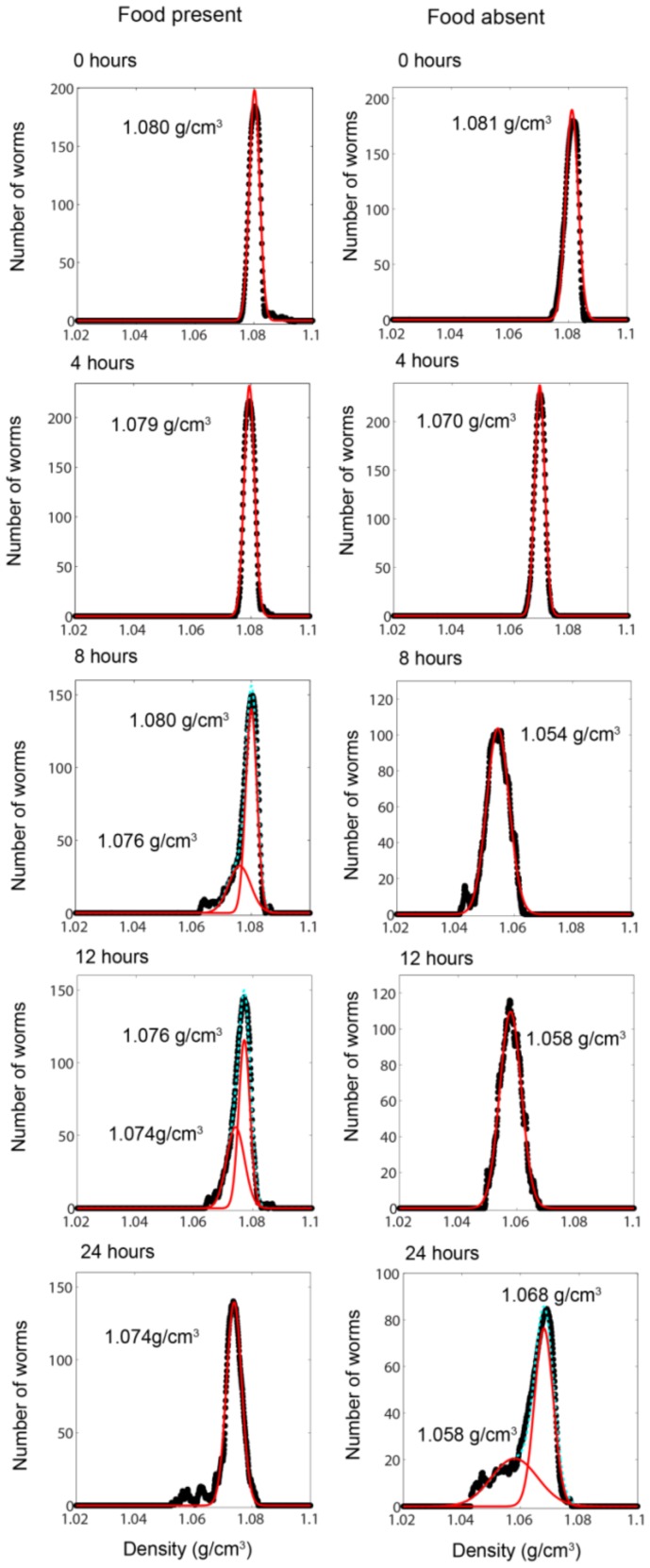
Changes in the distribution of mass densities of adult *C.elegans* subjected to caloric restriction. Each panel shows the distribution of densities of well-fed and starved worms at the indicated times. 0 hour corresponds to the time point immediately after the L4/adult molt. The red lines are Gaussian fits to the data and the numbers indicate the location of the peak(s) in the distributions.

Since the response of *C. elegans* under conditions of caloric restriction is of interest, we examined the effects of starvation on the density of the worms. We incubated synchronized populations of worms that had just completed the L4/adult molt on 20 agar plates devoid of food, and 20 agar plates with ample source of bacterial food. We harvested worms from three or four plates per time point and measured the density of the starving and well-fed worms at zero, four, eight, twelve, and twenty-four hours. Eggs or larval stages that were deposited onto the plates by the adults during culture were removed using the procedure described in the Material and Methods section and thus were not included in these measurements. **Figure 3** shows the density distribution curves of the worms cultured with ample food.

The mean density (calculated from the primary peak of the Gaussian fits) of the worms decreased from 1.080 g/cm^3^ at hour zero to 1.074 g/cm^3^ after twenty-four hours of aging. In some cases, such as for the measurements done at hour eight and hour twelve, the distribution of densities was asymmetric, with a longer tail at lower densities. The data in these cases were better fit with a double-peaked Gaussian distribution. The location of the secondary smaller peak at hour eight corresponded to the center of the primary peak at hour twelve, and the secondary peak at hour twelve corresponded to the center of the primary peak at hour twenty-four. There are two possible explanations for these findings. The first is that the smaller peak could reflect imperfect synchronization of the worms on the plates: a subpopulation of older worms was sampled in our population at hours eight and twelve. This sampling resulted in the secondary, smaller, peaks at these times. This explanation is supported by the observation that the secondary peaks correspond to primary peaks at the later sampling time. These secondary peaks were also present in replicate experiments, which perhaps reflect the difficulty of obtaining perfectly synchronized populations. An alternate explanation is that the worms show some unknown variation, perhaps due to differences in egg-laying behavior, which caused a broadening of the distributions of densities at hour eight and hour twelve of adulthood. Examination of the worms under the microscope did not show any noticeable differences in internal morphology or physical characteristics such as length or volume. Furthermore, the secondary peaks were not due to improper separation of larval stages from the sampled population, since larvae have a much higher density than the adults. It is apparent, however, that subpopulations within a general population of worms can be detected through measurements of density.

The distribution of densities of the worms is dramatically different for worms deprived of food. The mean density of adult worms under starvation stress decreased from 1.080 g/cm^3^ at hour zero to 1.070 g/cm^3^ at hour four. The mean density decreased further to 1.054 g/cm^3^ at hour eight and increased slightly to 1.058 g/cm^3^ at hour twelve. This increase in the mean density seemed to signify a change in the biological response of the worm because of continued food deprivation. Indeed, at twenty-four hours, the distribution of densities moved towards even higher values and peaked at 1.068 g/cm^3^ (**Fig. 3**). A significant secondary peak at 1.058 g/cm^3^ was also present at this time point.

The increase in density of most of the worms at twenty-four hours of starvation was initially puzzling. We propose an explanation based on the known response of wildtype adult worms to starvation stress [25], [40], and based on our observations of the density of larval stages of the worms. Prolonged starvation leads to egg-retention and development of larvae within the adults, i.e. the ‘bagging’ phenotype [25], [40] (also observed in our worms). These larvae use the parent body as a food source, consuming the adults from within before exiting the empty cuticle [25], [40]. Since the densities of larvae are significantly higher (**Fig. 2** **B**) than that of the adults, it is reasonable to expect that the net density of the adult worms will increase as the larvae developed within them. Worms recovered from the upper regions of the band (corresponding to the lower density secondary peak) showed less obvious evidence of bagging, when examined under the microscope, whereas worms recovered from the lower region of the band (corresponding to the higher density primary peak) had many larval worms that were motile within the parents (**Figure S2**). At this stage, it was unclear if the adults could be considered alive since the larvae had begun consuming the parents. After a further 24 hours of food deprivation, individual worms were distributed across a range of heights in the centrifugation media signifying that the population does not have a well-defined mean density (**Figure S3**). Thus, the evolution of the mass densities due to complete starvation in adult hermaphrodites could not be studied beyond 24 hours without chemical or genetic interventions, which is beyond the scope of this work. Worms that were deprived of food at the onset of the L4 larval stage, rather than as young adults, are apparently more resistant to the bagging phenotype, and can survive for more than 10 days under conditions of complete starvation [25].

Along with the dramatic changes in the mean density, the widths of the distributions, taken as the standard deviation of the primary Gaussian peak, were broader for worms starved for greater than four hours (**Fig. 3**). The difference in standard deviations of the Gaussian between worms with ample food and those that lacked food was, at 4 hours ∼0 g/cm^3^, 8 hours ∼0.003 g/cm^3^, 12 hours ∼0.003 g/cm^3^, 24 hours ∼0.003 g/cm^3^. This broadening of the distribution of densities is suggestive of the differences in adaptation to starvation stress in individual worms.

### Decrease in density correlated with lowering of glycogen content in food-deprived worms

**Figure 4.The:**
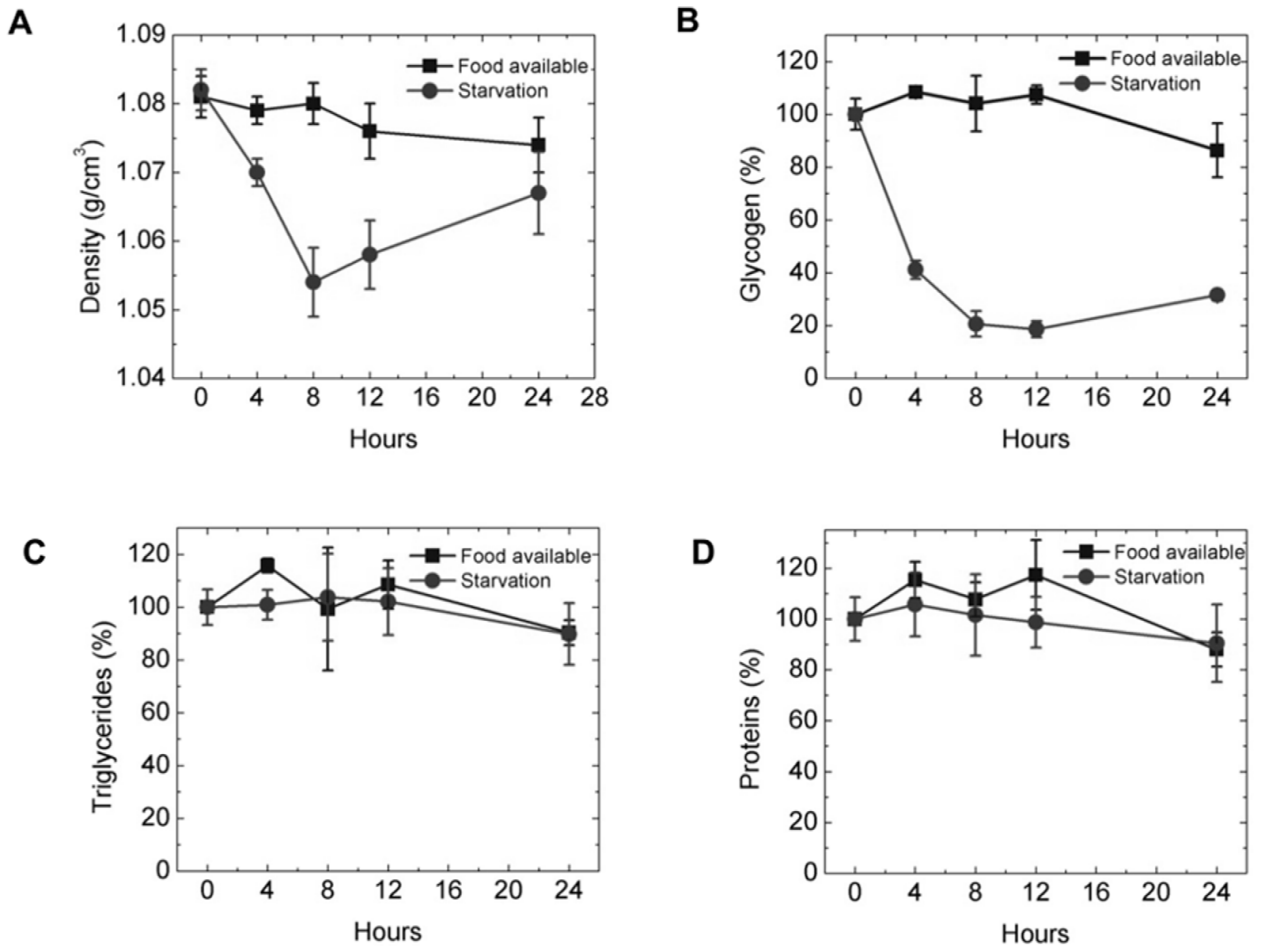
The change in density of starved worms correlates with the consumption of glycogen. (A) Densities of worms with ample food and under starvation. (B,C) Percentage of macronutrients—glycogen, triglycerides and proteins— normalized with respect to the values measured at t = 0 hours. The decrease in density correlated with the depletion of glycogen in the starved worms.

We conducted enzyme-based colorimetric assays on homogenates to quantify the relative protein, glycogen, and fat (glycerides) content of the worms to determine if we could model our observations of the changes in density of the worms in terms of gross changes in macronutrient composition. Since there is a clear shift in the biological response of the worms to facultative matricide after eight hours of starvation, it is unlikely that a simple compositional model can explain the increase in density in these worms, so we focus on the density of worms subject to 8 hours starvation. We find that the glycogen content of the worms, normalized by the glycogen content of well-fed worms at the same time point, falls by almost 50% during the first eight hours of starvation (**Fig. 4** **B**). This decrease in glycogen content correlated with the observed decrease in the density of the worms. The amount of proteins and triglycerides fluctuates without any apparent trend (**Fig. 4** **C, D**). Since the density of glycogen is relatively high (1.40–1.62 g/cm^3^) [43] it is rational that the density of the whole organism decreases upon consumption of glycogen (**Fig. 4** **A, B**).

We estimate the final density of the worm, *ρ^f^* due to changes in composition, using Equation 1. *ρ^o^* is the initial density of the worm, *f_i_* is fractional loss of constituent *i* based on initial mass of the worm, and 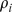 is the density of the constituent *i*, with *i* serving as an index denoting glycogen, fat, or protein (see **Text S1** for derivation).
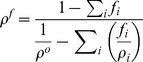
(Equation 1)Based on our compositional measurements, we took *f_fat_ = f_protein_ = 0*. From Eq. 1 the estimated density of the worm at hour four is1.072 g/cm^3^, which matched well with the density measured through centrifugation. At hour eight, we calculated an expected density of 1.070 g/cm^3^. This value is significantly higher than the measured value of 1.054 g/cm^3^. Our simplified model thus under-predicts the changes in density observed at hour eight (See **Figure S4**). Other physiological processes that are activated in response to starvation, such as changes in egg-laying patterns, are probably contributing to the observed changes in density. An alternate explanation for our observations is putative changes in water content in the worm; it is known that worms maintain osmotic balance by synthesizing glycerol under starvation stress [19], and that osmotic balance in dauers is maintained as long as lipid stocks are present [18]. Thus, it is unlikely that the changes in density that we observe during the initial stages of starvation (i.e. up to eight hours) are due to osmotic dysregulation. At the later stages of starvation, particularly when the worms begin to bag, it is more likely that systemic failures in homeostasis could lead to large changes in density due to influx or efflux of water.

Since it appeared that changes in glycogen content was the primary reason that the density of adult worms changed during the early stages of starvation, we wished to determine if the glycogen content, and hence the density of the worms, would change if feeding was resumed after a period of starvation. We thus measured the density and composition of worms that were fed after four hours of starvation. We observed that the density of the worms increased, and returned to pre-starvation values after eight hours of feeding (**Figure S5 A**). The glycogen lost due to starvation was restored once the worms were provided with food (**Fig. S5 B**). This observation is consistent with the view that glycogen depletion/storage decreases/increases the density of adult worms during the early stages of starvation.

### Density-based assay for detecting exposure of *C. elegans* to the antihelmetic drug Ivermectin

**Figure 5:**
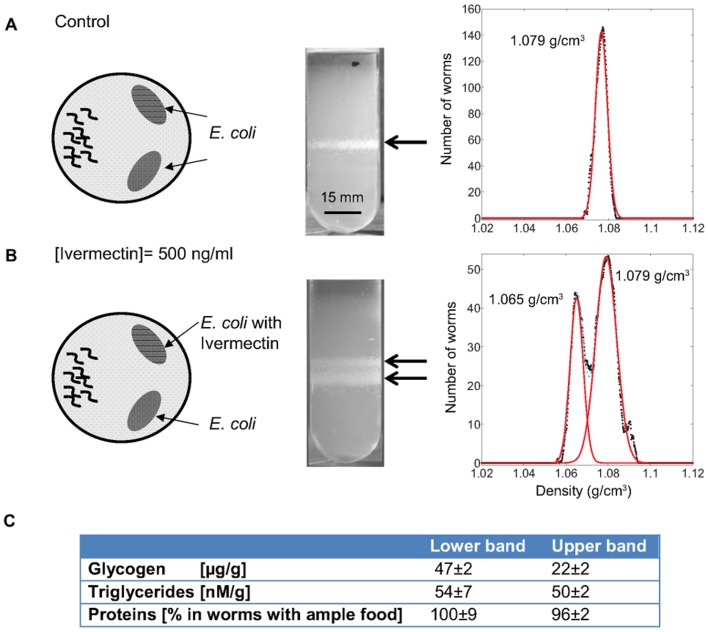
Demonstration of a density-based assay. (A) Worms grown on agar plates without drugs (control). The distribution of densities was unimodal. (B) Worms grown on agar plates with one lawn of bacteria containing Ivermectin. The distribution of densities after six hours was bimodal. Red lines are Gaussian fits to the data. (C) Composition of the worms extracted from the lower band (not exposed to drug) and upper band (exposed to drug). Worms in the lower band had approximately half the glycogen content of worms in the upper band, consistent with a starvation-like response.

Since early stage starvation is clearly detectable in *C. elegans* through measurements of density, we designed a density-based assay to detect the effects of drugs on the worms. As proof of principle, we administered sublethal doses of the antihelmetic Ivermectin (22,23-dihydroavermectin B1a+22,23-dihydroavermectin B1b, concentration 500 ng/mL, [32]) to the worms by spiking their OP50 *E. coli* food source. Ivermectin paralyzes the muscles of the worm. We deposited two volumes of *E. coli* suspension (500 µL) on two separate locations of an agar plate and allowed them to dry to form two lawns of bacteria. One lawn contained the drug and the other was free of the drug to serve as an internal control (**Figure 5**). We placed synchronized adult worms on the plates and cultured the worms on the plates for six hours at 24°C.

We hypothesized that worms consuming the drug-free bacteria should exhibit no change in density when compared to baseline values, while worms consuming the drug-doped bacteria should show a change in density if the drug caused a starvation-like response. We confirmed that worms on control plates with both lawns containing no drug showed a normal distribution of densities peaked at 1.079 g/cm^3^ (**Fig. 5** **A**). Worms incubated on plates with Ivermectin showed a clear bimodal distribution in densities (**Fig. 5** **B**), one peaked at 1.079 g/cm^3^ that corresponded to the peak in the control, and the other, a prominent peak at 1.065 g/cm^3^. Worms extracted from the band peaked at 1.079 g/cm^3^, which were immobile since they were cooled to 4°C for the measurement, and placed on agar plates with *E. coli* at 24°C resumed movement and feeding activity in ∼10 minutes. Worms extracted from the band peaked at 1.065 g/cm^3^ and placed on agar plates with *E. coli* at 24°C, took about 12 hours before resuming movement and feeding activity. Thus, we conclude that the worms with the lower densities were not able to feed due to exposure to Ivermectin. Colorimetric composition measurements of these worms confirmed that their glycogen content was lower than those of the worms from the band peaked at 1.079 g/cm^3^ (**Fig. 5** **C**). Note that although there were equal numbers of drug spiked and non-spiked *E. coli*, only 33 % of the worms exhibited effects consistent with exposure to the drug. Either this could reflect a natural variation in the resistance to the drug in the population, or it could reflect avoidance behavior of some of the worms to the drug-contaminated food source. It is likely that any process– for example exposure to drugs, genetic modification [2], or RNAi– that affects the metabolism of glycogen, egg-laying, and/or the development of bag-of-worm phenotypes will lead to changes in density that can be detected in a density-based assay. Furthermore, a greater degree of separation of populations of worms of differing densities can be obtained by preparing discontinuous steps in density, rather than a continuous gradient in the centrifugation media (**Text S2, Figure S6**).

## Conclusions

We have shown that isopycnic centrifugation of cold-immobilized worms in density gradients provides a measure of the distribution of densities in populations of hundreds of worms and that density is a convenient phenotypic readout in *C. elegans*. Due to the many factors that affect a systemic phenotype such as density, the information content obtained is low, but measurements of density is potentially a high-throughput batch screening mechanism that is conveniently applicable to large populations.

Although relatively little attention has been paid to the genetics of carbohydrate metabolism in *C. elegans* compared to the regulation of fat, our experiments reveal that the consumption of glycogen during early stages of starvation is a major biological adaptation to starvation stress in adult *C. elegans*. Moving forward, some obvious avenues of research to pursue include measuring the density of metabolic mutants, and measuring changes in the density of larval stages, males, or dauers that are subject to caloric restriction. A density-based screen of the drug Ivermectin demonstrates the convenience of screening for drugs on populations of worms grown on plates. This type of screen might be a useful tool for drug development using this organism as a target or as a model.

## Materials and Methods

### Culture and harvesting of worms

We use ‘wild-type’ worms N2 from the Caenorhabditis Genetic Center (University of Minnesota). We cultured and synchronized worms using well-established protocols. Briefly, we grew the worms at 24°C on agar plates (4 in. diameter) seeded with *E. coli* OP50 [44]. We harvested the worms by rinsing three to four plates (approximately 200 worms per plate) with phosphate buffered saline (PBS). The buffer containing the worms were collected in an Eppendorf centrifuge tube (2-mL). We separated the worms from the *E. coli* by allowing the worms to settle to the bottom of the centrifuge tube (about one minute). We then removed the supernatant, and resuspended the worms in clean PBS. We repeated this procedure at least 5 times to ensure removal of most of the bacteria. After cleaning the worms of bacteria, we removed any remaining eggs from the sample by passing the suspension through a nylon mesh with a mesh size of 25 µm (Small Parts). The worms, which were larger than the mesh openings, were retained on the mesh while the eggs passed through [28]. Washing the worms off the mesh with clean PBS into a clean Eppendorf tube was the final step of our harvesting procedure. A single harvesting took about 20 minutes, which was sufficient time for the worms to void their guts of feces from their last meal.

### Preparation of Percoll™ centrifugation media

We prepared continuous linear density gradients using the commercial density gradient media Percoll™, which is an aqueous suspension of ∼15 nm diameter colloidal silica covered with a stabilizing layer of the polymer polyvinylpyrrolidone. Percoll™ can be made isotonic throughout the gradient, and the local density in the gradient can be controlled by diluting neat Percoll with a liquid of lower density. We use a commercial gradient generator (Sigma-Aldrich, model number Z340413) and a peristaltic pump (Cole-Parmer) to generate linear gradients by mixing neat Percoll™ with neat PBS. The generator mixed the two solutions, while a peristaltic pump poured the resulting mixture into a 50-mL centrifuge tube at a constant rate to obtain continuous columns of liquid with a density of 1.123 g/cm^3^ at the bottom of the tube and 1.003 g/cm^3^ at the top of the tube. The gradient in density was 0.0023 g/cm^3^ per mm. We confirmed the reproducibility and the linearity of the gradient with calibration beads of known densities (**Figure 1** **A**).

### Procedure for measuring the distribution of densities of the worms

We immobilized the worms by cooling the worms to 4°C. At this temperature, all muscle movements stop and the worms became dormant. The worms were still alive, once brought back to room temperature the worms resumed movement and feeding activity. The cooled worms were gently introduced to the top most layer of pre-cooled density gradients with a micropipette and were centrifuged in a temperature controlled centrifuge set at 4°C at 1000× g for 20 minutes. After centrifugation, we moved the centrifuge tubes to a cold room set at a constant temperature of 4°C. The worms thus remained immobile throughout the experiment. In some cases, we transferred the worms using a glass transfer pipette from the density gradients into a 2-mL Eppendorf tube, for further enzyme-based compositional analysis.

### Data acquisition and analysis

Still images of the worms in the gradients were captured with a Nikon D5100 digital camera equipped with a ring flash to obtain even illumination. We were careful to use identical settings (lighting, exposure) for all our experiments. We subtracted the background of each image in the centrifugation media using the image processing software ImageJ (NIH, Bethesda). A square region of interest was drawn along the long axis of the centrifuge tube and an intensity profile was calculated. The intensity data was then normalized in Matlab (Mathworks, v. 7.1) by setting the area under the curve to one. The number of worms in the distribution was estimated by comparing intensities of calibration images with known numbers of worms in the centrifuge media. To simplify the analysis, we consider the distribution of the worm densities in a population as being continuous. A continuous distribution allows for fractional number of worms in each density bin. Single or double-peaked Gaussian distributions were fitted to the normalized data using the curve-fitting function ‘cftool’ in Matlab.

### Preparation of the worms for enzymatic compositional analysis

Worms were collected in 2-mL centrifuge tubes and rinsed briefly with ultrapure water to remove salts from the media. We killed the worms rapidly by plunging the tubes into a bath of liquid nitrogen for 30 seconds. We then freeze-dried the frozen pellet of worms for two hours in a lyophilizer. Following the manufacturers' directions, we mixed four milligrams of freeze-dried worms with 500 µL of surfactant solution (5% v/v IGEPAL® or octylphenoxypolyethoxyethanol) in a centrifuge tube. The surfactant disrupts lipid membranes and helps solubilize fat. We added 10–20 glass beads (1 mm diameter) to the centrifuge tube and agitated the tube in a vortex mixer at maximum power for 10 minutes. We then diluted the resulting homogenate 10 times with ultrapure water to obtain a final mass concentration of 0.8 mg/mL. The homogenate was centrifuged at 14,000× g for five minutes to pellet insoluble material, and the supernatant was saved for further analysis.

### Quantifying the composition of the worms

We analyzed the clarified homogenate to determine the concentration of metabolic macromolecules. We used commercial colorimetric assays to quantify proteins (Bio-Rad, catalog no. 500-0111), glycogen (Abcam®, catalog no. ab656260) and triglycerides (BioVision, catalog no. K622-100). The assays were performed in polystyrene 96-well plates, according to the respective manufacturers' protocols, and quantified with a plate reader (Spectramax M2e). We confirmed that the surfactant IGEPAL that we used to dissolve the worms did not interfere with the assays.

In the protein assay, proteins reacted with copper in an alkaline medium. Folin reagent (sodium 1,2-naphthoquinone-4-sulfonate) reduces in the presence of the copper-treated proteins, and produced one or more of several possible reduced species which have a characteristic blue color. Absorbance was measured at 750 nm. In the glycogen assay, the enzyme glucoamylase hydrolyzed the glycogen to glucose. Products from glucose oxidation reacted with a colorimetric probe. The change in color was measured by absorbance at 570 nm. In the triglyceride assay, triglycerides were broken down to free fatty acids and glycerol by a phospholipase. The glycerol was oxidized to generate a product that reacted with the colorimetric probe. The change in color was measured by absorbance at 570 nm.

### Starvation assays of the worms

Worms cultured, synchronized, and harvested employing the procedure described above, were introduced onto agar plates devoid of a food source. The plates were placed in an incubator at 24°C for four, eight, twelve and twenty-four hours. The worms were collected using the same procedure described above after the designated starvation period for analysis.

### Density-based drug screens on the worms

We spiked *E. coli* with Ivermectin (500 ng/mL) by adding 10 µL of an Ivermectin stock solution (50 µg/mL in ethanol) to each milliliter of *E. coli*. We deposited two lawns of *E. coli* on each of the agar plates. One lawn contained drug free *E. coli* and the second, *E. coli* with Ivermectin. We deposited adult worms on the plates away from either of the lawns. The worms foraged at both lawns, thus a fraction of the population in each plate was exposed to the drug. We incubated the worms on the plates for six hours at 24°C before harvesting and analysis.

## Supporting Information

Figure S1 **Image showing** ***C. elegans*** **eggs in the centrifugation media.** Eggs have a lower density than adults and larvae. Detritus from the culture that could not be separated can be seen in the centirfuge media as a diffuse layer above the well-defined band of eggs (arrow).
(TIFF)

Figure S2 **Micrograph of worms starved for 24 hours showing the bag-of-worms phenotype.** Adult hermaphrodites experiencing prolonged starvation retain their eggs within their bodies. The eggs hatch and develop into larvae that consume the adults from within. The optical micrograph here shows L1 larvae within the adult bodies.
(TIFF)

Figure S3 **Adult N2 worms starved for more than 24 hours do not show well-defined peaks in the distribution of densities.** The photograph demonstrates that the worms settle in a diffuse layer in the centrifuge media.
(TIF)

Figure S4 **Measured densities for fed and starved worms and the expected density of the starved worms calculated using compositional data and** **Eq. 1** **.**
(TIF)

Figure S5 **Density and glycogen lost is recovered when worms are fed after a short period starvation.** (A) Densities determined for worms fed after 4 hours of starvation. (B–D) Percentage of macronutrients—glycogen, triglycerides and proteins—relative to the concentration at t = 0. Glycogen lost during starvation was restored upon feeding.
(TIF)

Figure S6 **Density measurements of well-fed and starved worms in Percoll™ centrifuge media with discontinuous steps in density.** Each 7 mm layer of centrifuge media is isodense. The step in density between the layers is 0.005 g/cm^3^. It is apparent that starved and non-starved worms have very different densities and that two populations of worms can be resolved in worms subject to starvation; a primary population at the interface between the 1.070–1.065 g/cm^3^ layer, and a secondary population at thse interface between the 1.065–1.060 g/cm^3^ layer.
(TIF)

File S1 Text S1. Derivation of Equation 1. Text S2. Method used to prepare Percoll™ centrifugation media with discontinuous steps in density.
(PDF)
