# Supplementary figures and images for: Shifts in the Distribution of Mass Densities Is a Signature of Caloric Restriction in Caenorhabditis elegans
Source: PLoS One. 2013 Jul 29;8(7):e69651. doi: 10.1371/journal.pone.0069651 (PMC3726776; doi:10.1371/journal.pone.0069651)

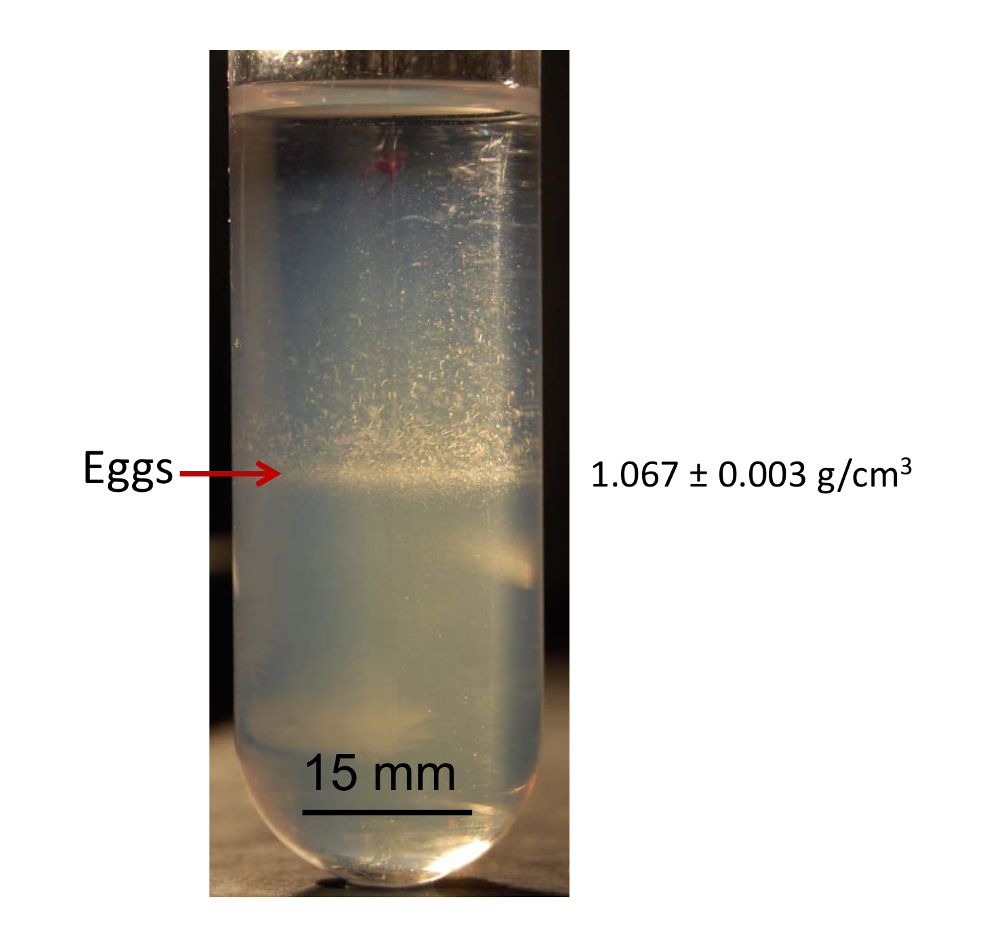

Supplement: Figure S1 — Image showing C. elegans eggs in the centrifugation media. Eggs have a lower density than adults and larvae. Detritus from the culture that could not be separated can be seen in the centirfuge media as a diffuse layer above the well-defined band of eggs (arrow). (TIFF) [file pone.0069651.s001.tiff]

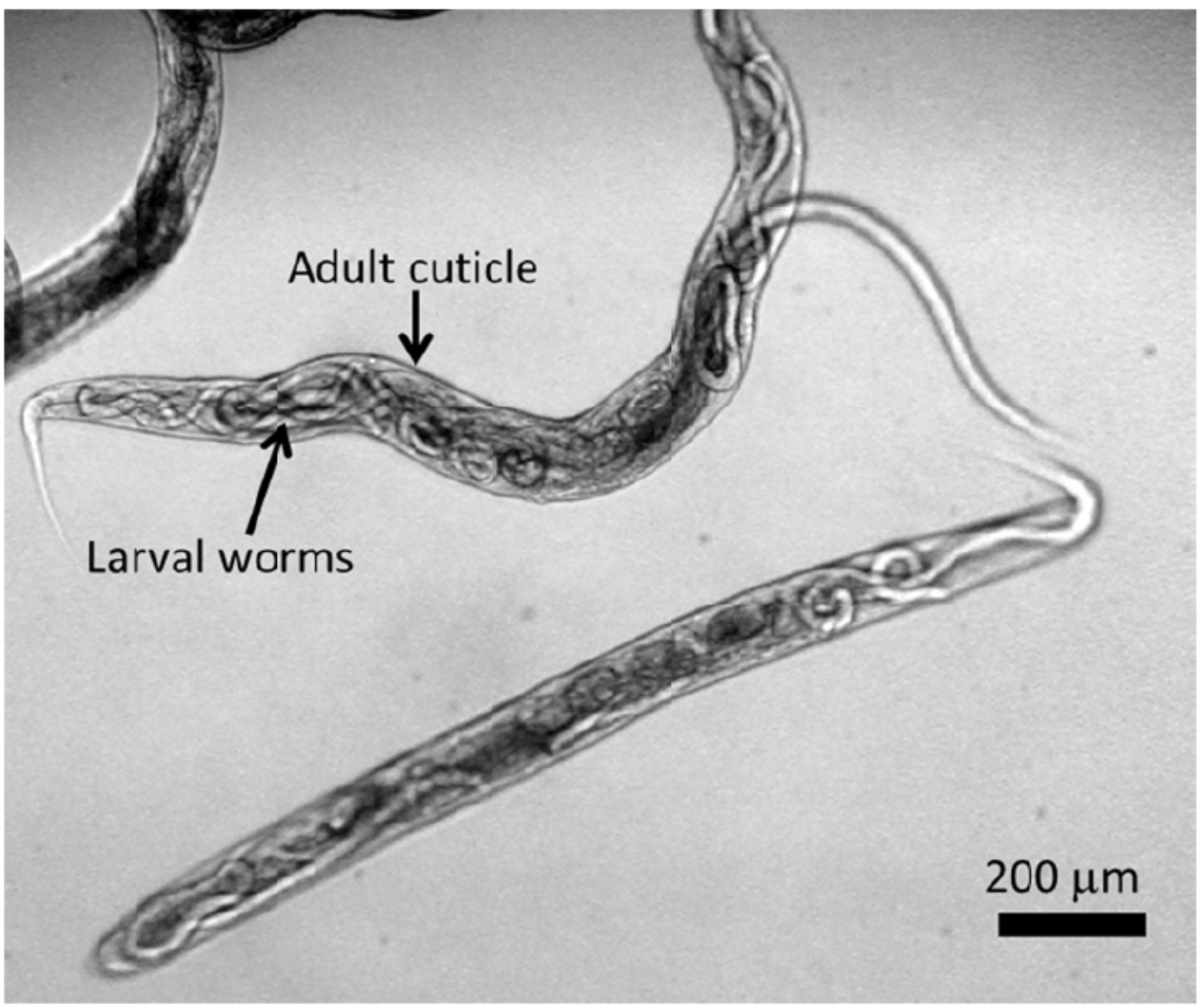

Supplement: Figure S2 — Micrograph of worms starved for 24 hours showing the bag-of-worms phenotype. Adult hermaphrodites experiencing prolonged starvation retain their eggs within their bodies. The eggs hatch and develop into larvae that consume the adults from within. The optical micrograph here shows L1 larvae within the adult bodies. (TIFF) [file pone.0069651.s002.tiff]

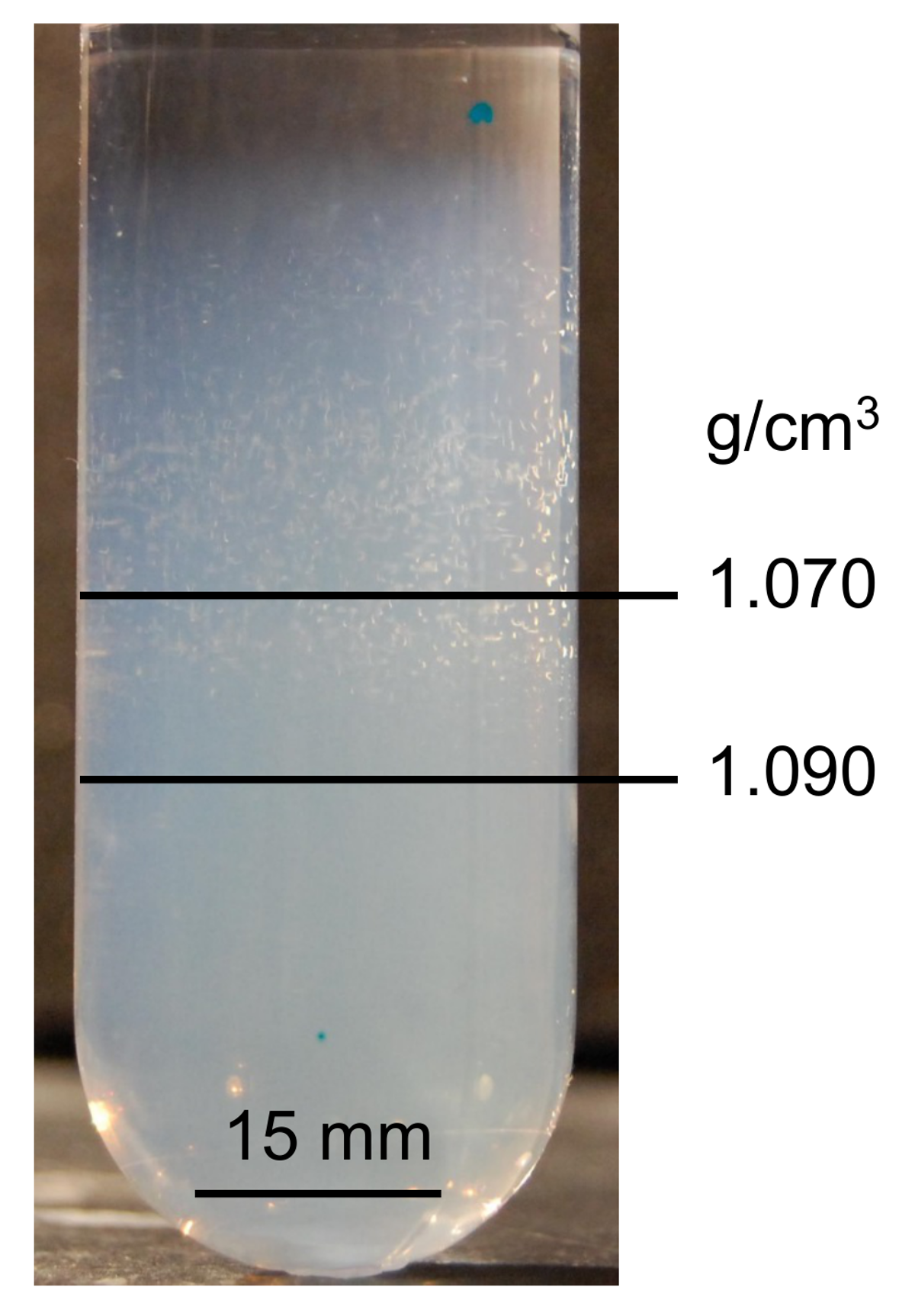

Supplement: Figure S3 — Adult N2 worms starved for more than 24 hours do not show well-defined peaks in the distribution of densities. The photograph demonstrates that the worms settle in a diffuse layer in the centrifuge media. (TIF) [file pone.0069651.s003.tif]

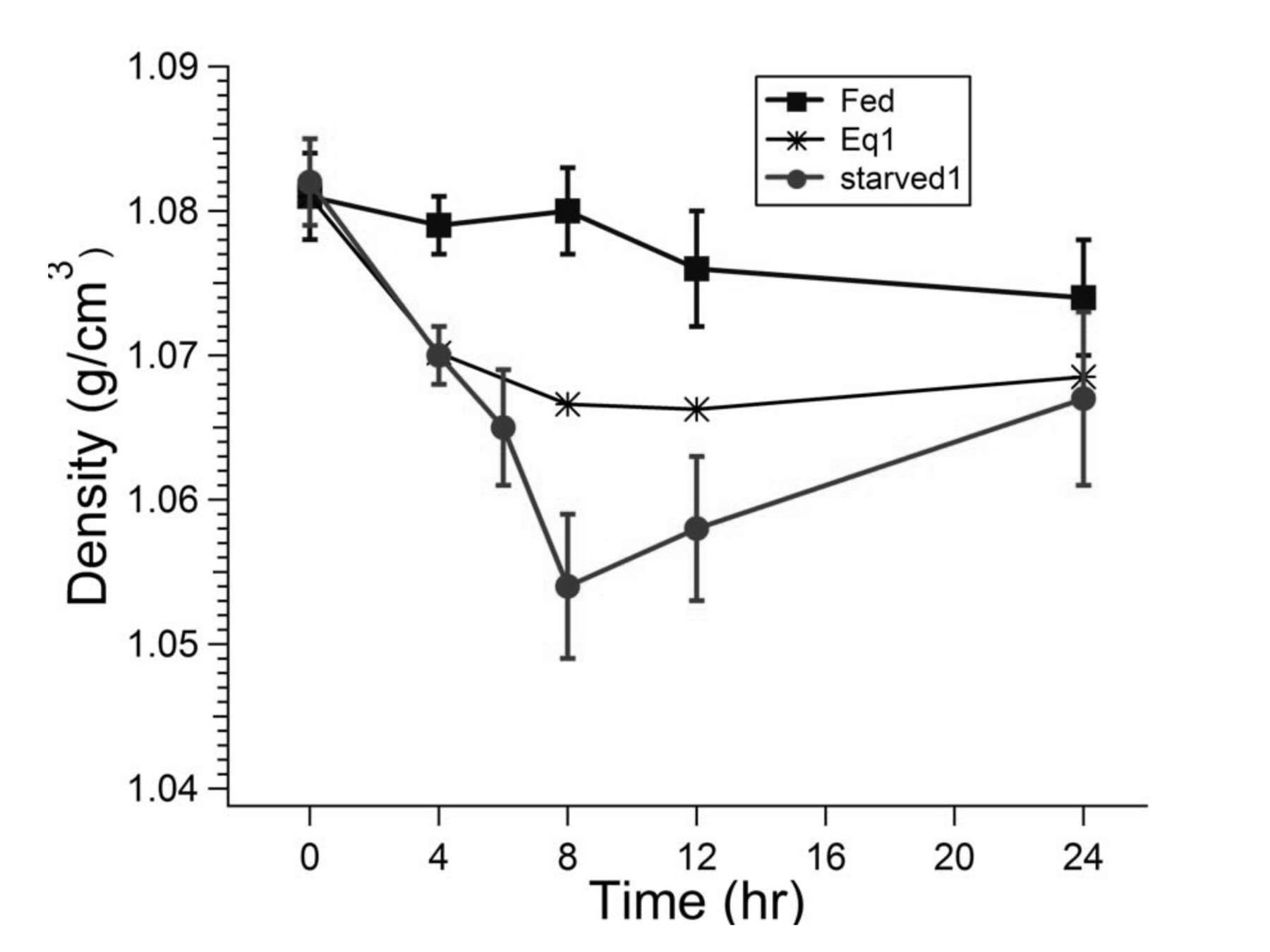

Supplement: Figure S4 — Measured densities for fed and starved worms and the expected density of the starved worms calculated using compositional data and Eq. 1 . (TIF) [file pone.0069651.s004.tif]

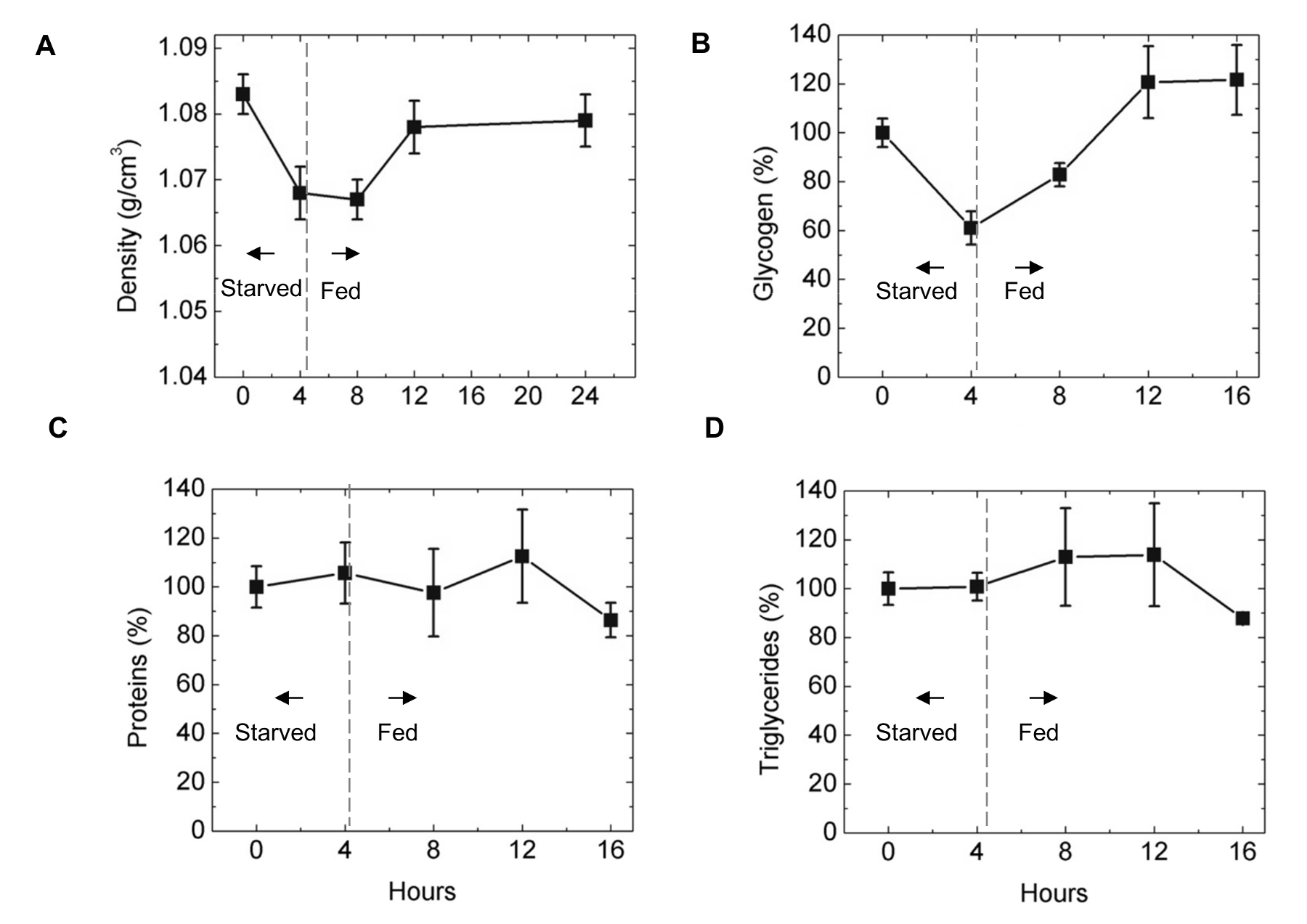

Supplement: Figure S5 — Density and glycogen lost is recovered when worms are fed after a short period starvation. (A) Densities determined for worms fed after 4 hours of starvation. (B–D) Percentage of macronutrients—glycogen, triglycerides and proteins—relative to the concentration at t = 0. Glycogen lost during starvation was restored upon feeding. (TIF) [file pone.0069651.s005.tif]

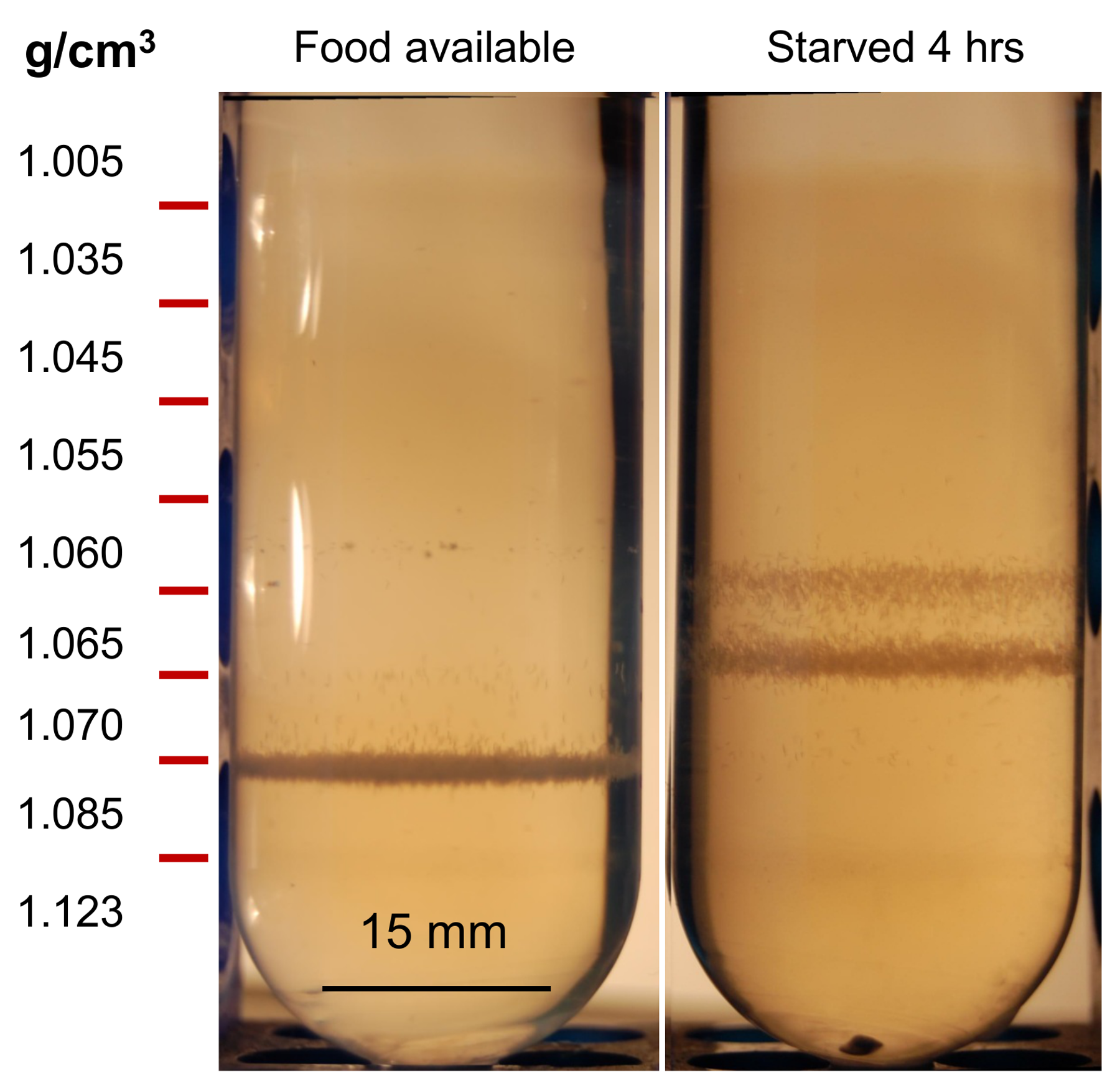

Supplement: Figure S6 — Density measurements of well-fed and starved worms in Percoll™ centrifuge media with discontinuous steps in density. Each 7 mm layer of centrifuge media is isodense. The step in density between the layers is 0.005 g/cm3. It is apparent that starved and non-starved worms have very different densities and that two populations of worms can be resolved in worms subject to starvation; a primary population at the interface between the 1.070–1.065 g/cm3 layer, and a secondary population at thse interface between the 1.065–1.060 g/cm3 layer. (TIF) [file pone.0069651.s006.tif]
