# Supplementary material for: Shifts in the Distribution of Mass Densities Is a Signature of Caloric Restriction in Caenorhabditis elegans
Source: PLoS One. 2013 Jul 29;8(7):e69651. doi: 10.1371/journal.pone.0069651 (PMC3726776; doi:10.1371/journal.pone.0069651)
Supplement: File S1 — Text S1. Derivation of Equation 1. Text S2. Method used to prepare Percoll™ centrifugation media with discontinuous steps in density. (PDF) [file pone.0069651.s007.pdf]

Text S1: Expected change in density due to changes in the composition of the worms

The initial mass of the worm m^o is the sum of mass of its constituents. Using the indices fat (f), water (w), protein (p), glycogen (g) and others (water, salts, dissolved gases etc.) (q) and the superscript o to denote the initial state we can write

$$m_o = m_f^o + m_p^o + m_g^o + m_w^o + m_q^o = \sum_i m_i^o, \tag{1}$$

where the index i refers to a specific constituent. The specific volume, $v_i \equiv 1/\rho_i$ of each constituent i does not change for incompressible materials. The initial volume is then

$$V^o = \sum_i m_i^o v_i = \sum_i \left(\frac{m_i^o}{\rho_i}\right),$$

and the initial density of the worm is

$$\rho^o = \frac{1}{V^o}\sum_i m_i^o = \left(\frac{\sum_i m_i^o}{\sum_i \left(\frac{m_i^o}{\rho_i}\right)}\right).$$

The worm can either lose or gain constituents, with the incremental change in mass of each constituent i being Δm_i . The net change in the mass of the worm is

$$\Delta m = \sum_i \Delta m_i.$$

The final volume of the worm is related to the change in mass, initial mass, and the specific volumes

$$V^f = \sum_i \left(\frac{m_i^o - \Delta m_i}{\rho_i}\right) = V^o - \sum_i \left(\frac{\Delta m_i}{\rho_i}\right),$$

yielding the expression for the final density

$$\rho^f = \left(\frac{m^o - \sum_i \Delta m_i}{V^o - \sum_i \left(\frac{\Delta m_i}{\rho_i}\right)}\right) = \rho^o \left(\frac{1 - \sum_i \frac{\Delta m_i}{m^o}}{1 - \frac{1}{V^o}\sum_i \left(\frac{\Delta m_i}{\rho_i}\right)}\right) = \left(\frac{1 - \sum_i \frac{\Delta m_i}{m^o}}{\frac{1}{\rho^o} - \sum_i \left(\frac{\Delta m_i/m^o}{\rho_i}\right)}\right) \tag{2}$$

To complete the derivation, we define the change in mass fraction of a constituent f_i as

$$f_i = \Delta m_i / m^o,$$

$$\rho^f = \left(\frac{1 - \sum_i f_i}{\frac{1}{\rho^o} - \sum_i \left(\frac{f_i}{\rho_i} \right)} \right). \tag{3}$$

Text S2: Preparation of Percoll^{TM} centrifugation media with discontinuous steps in density

We prepared solutions of Percoll^{TM} and phosphate buffered saline (PBS) with densities of 1.123, 1.085, 1.080, 1.075, 1.070, 1.065, 1.060, 1.055, 1.050, 1.045, 1.040, 1.035 and 1.003 g/cm^3 according to instructions from the manufacturer (Percoll: Methodology and Applications, Amersham Biosciences). We measured the densities of the solutions using a portable density meter (Model DMA 35, Anton Paar). Centrifugation media with steps in density were prepared by carefully layering (using a 5-mL syringe and a 15G needle (Becton Dickinson)) three milliliters each of the Percoll^{TM} mixtures of different densities into a 50-mL centrifuge tube. We introduced the layer with the highest density (1.123 g/cm^3) at the bottom of the tube, on top of which we layered consecutively mixtures of Percoll^{TM} with lower densities, ending with the least dense mixture (1.003 g/cm^3). Sharp jumps in density were thus established in the medium where the layers met. The steps in density were stable for at least 24 hours, though we typically used the centrifugation media within one hour of preparation. Centrifugation of the worms in these stepped density media were performed using similar protocols to those used for centrifugation in continuous density gradients.
